# Functional and differential proteomic analyses to identify platelet derived factors affecting ex vivo expansion of mesenchymal stromal cells

**DOI:** 10.1186/1471-2121-14-48

**Published:** 2013-10-30

**Authors:** Sven Kinzebach, Lisa Dietz, Harald Klüter, Hermann-Josef Thierse, Karen Bieback

**Affiliations:** 1 Institute of Transfusion Medicine and Immunology, Medical Faculty Mannheim, Heidelberg University; German Red Cross Blood Service Baden-Württemberg, Friedrich-Ebert-Str. 107, Mannheim, Hessen D-68167, Germany; 2 Laboratory of Immunology & Proteomics, Department of Dermatology, University Medical Center Mannheim, Heidelberg University, Theodor-Kutzer Ufer 1, Mannheim D-68167, Germany; 3 Functional Proteome Analysis, German Cancer Research Center, Heidelberg, Germany; 4 Current address: Leibniz-Institut für Analytische Wissenschaften - ISAS - e.V, Otto-Hahn-Str. 6b, Dortmund 44227, Germany; 5 Current address: German Federal Institute for Risk Assessment, Max-Dohrn-Str. 8-10, Berlin 10589, Germany

**Keywords:** Bone marrow, Differential proteomics, Mesenchymal stromal cells, 2D-DIGE, Mass spectrometry, Platelets

## Abstract

**Background:**

Multilineage differentiation, immunomodulation and secretion of trophic factors render mesenchymal stromal cells (MSC) highly attractive for clinical application. Human platelet derivatives such as pooled human platelet lysate (pHPL) and thrombin-activated platelet releasate in plasma (tPRP) have been introduced as alternatives to fetal bovine serum (FBS) to achieve GMP-compliance. However, whereas both pHPL and tPRP support similar proliferation kinetics of lipoaspirate-derived MSC (LA-MSC), only pHPL significantly accelerates bone marrow-derived MSC (BM-MSC) expansion. To identify functionally bioactive factors affecting ex vivo MSC expansion, a differential proteomic approach was performed and identified candidate proteins were evaluated within a bioassay.

**Results:**

Two dimensional difference gel electrophoresis (2D-DIGE), MALDI-TOF analyses and complementary Western blotting revealed 20 differential protein species. 14 candidate proteins occured at higher concentrations in pHPL compared to tPRP and 6 at higher concentrations in tPRP. The candidate proteins fibrinogen and apolipoprotein A1 differentially affected LA- and BM-MSC proliferation.

In a second set of experiments, reference cytokines known to foster proliferation in FBS were tested for their effects in the human supplements. Interestingly although these cytokines promoted proliferation in FBS, they failed to do so when added to the humanized system.

**Conclusions:**

The differential proteomic approach identified novel platelet derived factors differentially acting on human MSC proliferation. Complementary testing of reference cytokines revealed a lack of stimulation in the human supplements compared to FBS. The data describe a new coherent approach to combine proteomic technologies with functional testing to develop novel, humanized, GMP-compliant conditions for MSC expansion.

## Background

Mesenchymal stromal cells (MSC) offer great potential for therapeutic application since they combine a number of biological properties such as multilineage differentiation, stromal support, immunomodulation, and secretion of trophic factors [1-3]. Due to the low frequency within tissues, in general ex vivo expansion is required to achieve a clinically-relevant cell dose. This has to comply with good manufacturing practice (GMP) guidelines. Fetal bovine serum (FBS) used in many protocols, however, is critically rated by the regulatory authorities due to the possible transmission of extraneous agents as well as the risk of triggering host immune responses comprising the therapeutic success [4-7]. A chemically-defined medium sufficiently mimicking serum compounds to provide growth and attachment factors, buffering and detoxifying agents, is still under development [5,8,9]. Currently human supplements, including platelet derivatives and human serum from autologous, allogeneic or cord blood sources, are assessed in pre- and clinical studies to replace FBS [8,9]. We have previously demonstrated that pooled human serum (HS), human platelet lysate (pHPL) and thrombin-activated platelet releasate in plasma (tPRP) are promising alternatives to FBS and support the main characteristics of MSC [10-13]. Interestingly, HS, tPRP and pHPL all promoted the proliferation of MSC from adipose tissue (lipoaspirate, LA-MSC) to a comparable extent [13]. However, bone marrow-derived MSC (BM-MSC) proliferation was significantly enforced solely by pHPL [11]. The different proliferative responses raised three questions: i) which bioactive proteins differ in the lysate and the releasate, ii) do these proteins exert different effects on LA- and BM-MSC, and (iii) can we extrapolate these findings to optimize chemically-defined MSC media?

Human serum and platelet granules contain various growth factors capable of promoting cell proliferation and tissue regeneration [8]. Identification and characterization of these factors was markedly achieved by bioinformatic approaches integrating proteomic data sets from plasma, serum, the entire platelet proteome and specific subproteomes with functional data [14-18]. These analyses have provided a comprehensive list of platelet and plasma proteins and as such contributed significantly to our current biomolecular understanding of these components and their function in the human body. They support a systems biology view on platelet protein function, network modules and enable evaluation of upcoming data sets [19,20].

Aiming to answer our questions we analyzed pHPL and tPRP with a differential proteomic approach and related the data to an in-depth catalog of human platelet proteins [20]. Upon integrating our own data with existing datasets on platelet factors differentially released upon activation [18,20-23], resulting potential bioactive proteins were functionally assessed on LA- and BM-MSC in vitro proliferation.

In addition, to validate our system we assessed the effects of selected reference cytokines. We chose cytokines described to enhance the proliferation of MSC in the presence of FBS asking whether these exert similar or different effects when added to pHPL or tPRP supplement.

## Results and discussion

### Bioactive factors promoting MSC expansion

**Figure 1:**
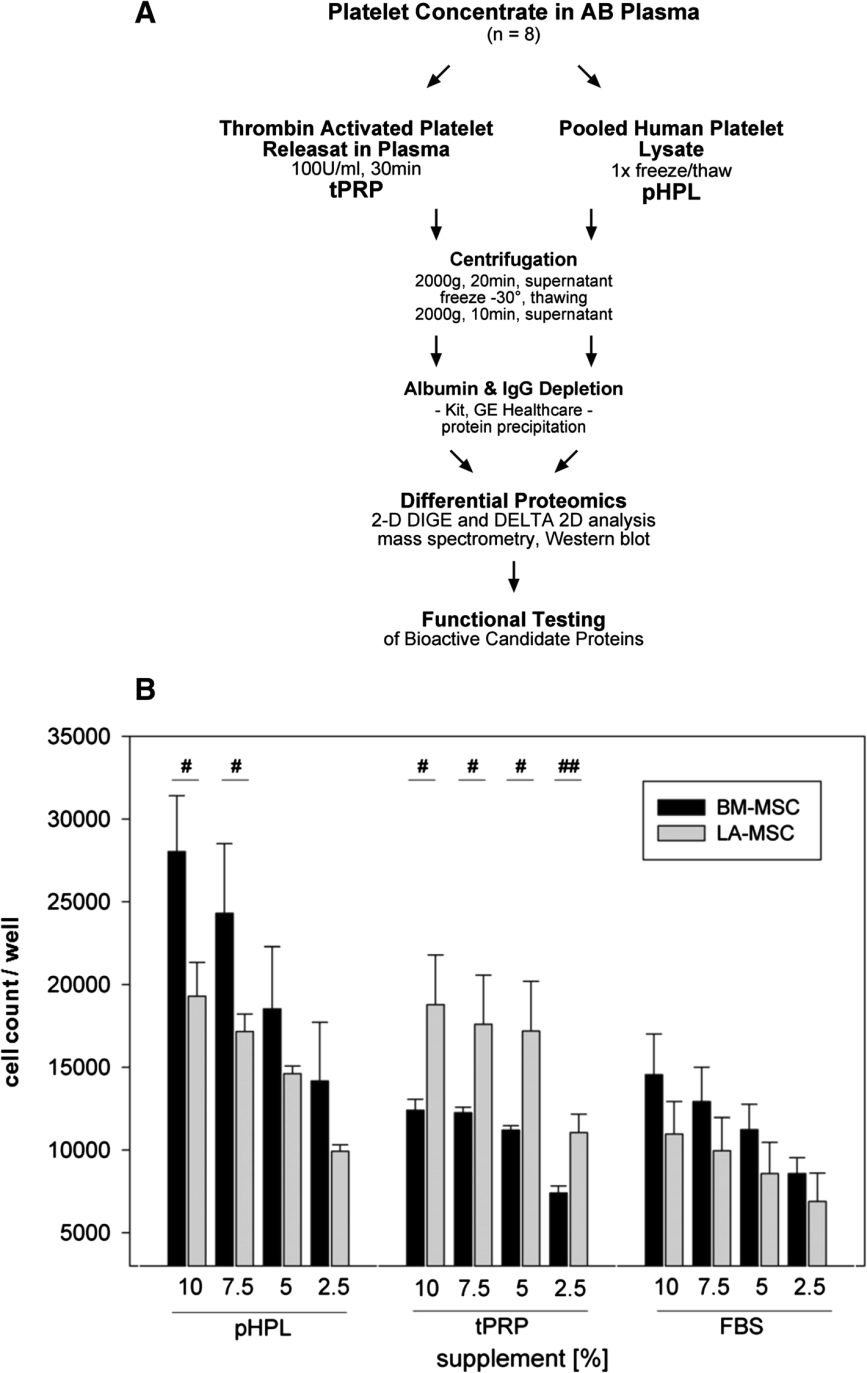
**Experimental strategy and effect of human supplements on MSC proliferation. (A)** Experimental strategy and **(B)** proliferation of MSC affected by the use of different supplements. Cell numbers were determined by the CellTiter-Glo assay on day 3 after seeding 3000 cells/well. Absolute MSC cell counts are shown related to decreasing supplement concentrations of pHPL, tPRP or FBS. Three independent experiments with three replicates were performed (#: p < 0.05; ##: p < 0.01).

Human platelet derivates support the expansion of MSC from different tissues without changing the differentiation capacity and immunoregulatory properties when compared to FBS supplementation (Additional file 1: Figure S1) [11,13,24,25]. Confirming previous results, pHPL-supplemented medium significantly promoted BM-MSC proliferation compared to tPRP- and FBS (Figure 1B, p < 0.05) [11]. In contrast, pHPL and tPRP accelerated the proliferation of LA-MSC in a similar way when compared to FBS (p < 0.05) [13]. The specifically enhanced proliferative response of BM-MSC towards pHPL was dose-dependent, with 2.5% pHPL comparable to 10% tPRP or 10% FBS. Interestingly, in tPRP dose dependency was not obvious within the range of 10-5%. Evaluating different batches, there were no significant differences in the proliferative response (not shown). These data clearly indicate that pHPL and tPRP contain different bioactive factors which specifically affect BM-MSC proliferation.

### Mass spectrometric identification of differential proteins in pHPL and tPRP

**Figure 2:**
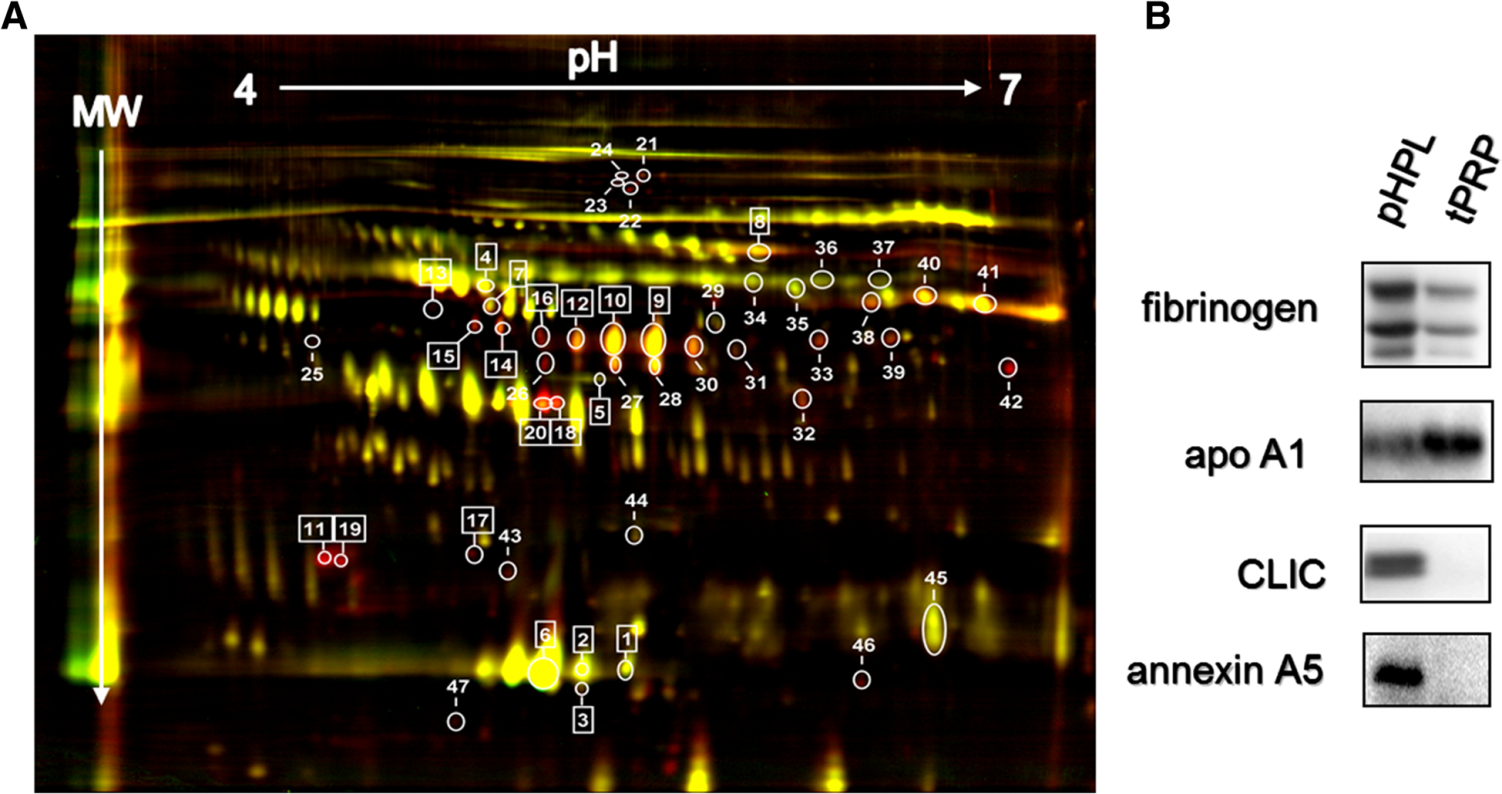
**Proteomics and western blot verification. (A)** Differential 2D-DIGE proteome map of pHPL and tPRP platelet derivatives containing bioactive factors. Differential proteins spots (a total of 47) were detected by utilizing strips from pH 4 to 7 and quantitative Delta 2D imaging software (t-test p ≤ 0.05). Proteins identified by mass spectrometry are specifically shown (no. in rectangle). **(B)** Complementary Western blot verification of differential proteins in albumin and IgG depleted pHPL and tPRP samples. Different protein concentrations were loaded: α-fibrinogen (2.5 μg protein/lane), α-apoA1 (2.5 μg protein/lane), α-CLIC (5 μg protein/lane), α-annexin A5 (30 μg protein/lane).

To detect and identify bioactive factors in pHPL and tPRP, a differential proteomic 2D-DIGE approach was performed (Figure 1A). After labelling pHPL and tPRP with CyDyes and subsequent 2D separation, 47 differentially occurring proteins were quantified by Delta 2D imaging software (Figure 2A). Selected differential proteins of pHPL and tPRP were picked from the preparative gel (Additional file 2: Figure S2) and subjected to MALDI-TOF analysis. In total, 19 plus one differential protein species were identified (Table 1, Figure 2A, Additional file 2: Figure S2). Six proteins appeared higher in tPRP whereas 14 proteins were more abundant in pHPL. One protein out of 20, Annexin A5, was not satisfactory identified by MS (5.6% below scoring) and initially not considered as identified, but subsequent Western blot analysis confirmed differential expression (Table 1, Figure 2B).

**Table 1:** **MS analysis of 20 (19) differential protein spot species in pHPL and tPRP**^**a) b)**^

**Ratio ‘pHPL’/‘tPRP’**	**Spot**	**Protein name**	**SwissProt ID**	**Sequence coverage (%)**	**Peptides queries matched**	**Percental match (%)**	**MOWSE score (> 56; p < 0.05)**	**MW (Da)**	**pI**
0.62	1	Apolipoprotein A-I	sp|P02647|APOA1_HUMAN	80	29	33.7	292	30759	5.56
0.65	2	Apolipoprotein A-I	sp|P02647|APOA1_HUMAN	64	23	26.4	205	30759	5.56
0.69	3	Retinol-binding protein 4	sp|P02753|RET4_HUMAN	67	11	15.3	114	23337	5.76
0.71	4	Angiotensinogen	sp|P01019|ANGT_HUMAN	19	9	14.8	62	53406	5.87
0.71	5	CD5 antigen-like	sp|O43866|CD5L_HUMAN	28	8	9	66	39603	5,28
0.74	6	Apolipoprotein A-I	sp|P02647|APOA1_HUMAN	59	23	29.5	193	30759	5.56
1.55	7	Vitamin D-binding protein	sp|P02774|VTDB_HUMAN	37	15	35.7	147	54526	5.40
1.84	8	Hemopexin	sp|P02790|HEMO_HUMAN	25	9	10.1	68	52385	6.55
2.68	9	Fibrinogen gamma chain	sp|P02679|FIBG_HUMAN	59	21	25.3	201	52106	5.37
2.77	10	Fibrinogen gamma chain	sp|P02679|FIBG_HUMAN	56	21	23.1	226	52106	5.37
2.98	11	Tropomyosin alpha-4 chain	sp|P67936|TPM4_HUMAN	33	10	17.2	83	28619	4.67
3.35	12	Fibrinogen gamma chain	sp|P02679|FIBG_HUMAN	27	13	15.1	88	52106	5.37
4.30	13	Tubulin beta chain	sp|P07437|TBB5_HUMAN	39	17	20.7	149	50095	4.78
4.59	14	Fibrinogen gamma chain	sp|P02679|FIBG_HUMAN	56	21	24.1	235	52106	5.37
6.00	15	Fibrinogen gamma chain	sp|P02679|FIBG_HUMAN	51	19	22.1	189	52106	5.37
6.69	16	Fibrinogen gamma chain	sp|P02679|FIBG_HUMAN	27	11	16.7	73	52106	5.37
10.36	17	Chloride intracellular channel protein 1	sp|O00299|CLIC1_HUMAN	43	9	18	88	27248	5.09
10.76	18	Actin, cytoplasmic 1	sp|P60709|ACTB_HUMAN	53	16	22.2	134	42052	5.29
11.01	19^b)^	Annexin A5	sp|P08758|ANXA5_HUMAN	21	5	10.4	53^b)^	35971	4.94
12.27	20	Actin, cytoplasmic 1	sp|P60709|ACTB_HUMAN	52	18	27.3	163	42052	5.29

^a)^Mascot software analysis by using SwissProt data base. Ratio pHPL/tPRP: <1 higher abundant in tPRP, >1 higher abundant in pHPL. PMF criteria: human source, one missed tryptic cleavage, methionine oxidation, score limit >56. ^b)^Despite the fact that potential MS identification of Annexin A5 is below required scoring (5.6% less), this non-significant MS hit was included into the list because the suggested high ratio and protein ID was confirmed by Western blotting.

### Verification of selected differential proteins

**Figure 3:**
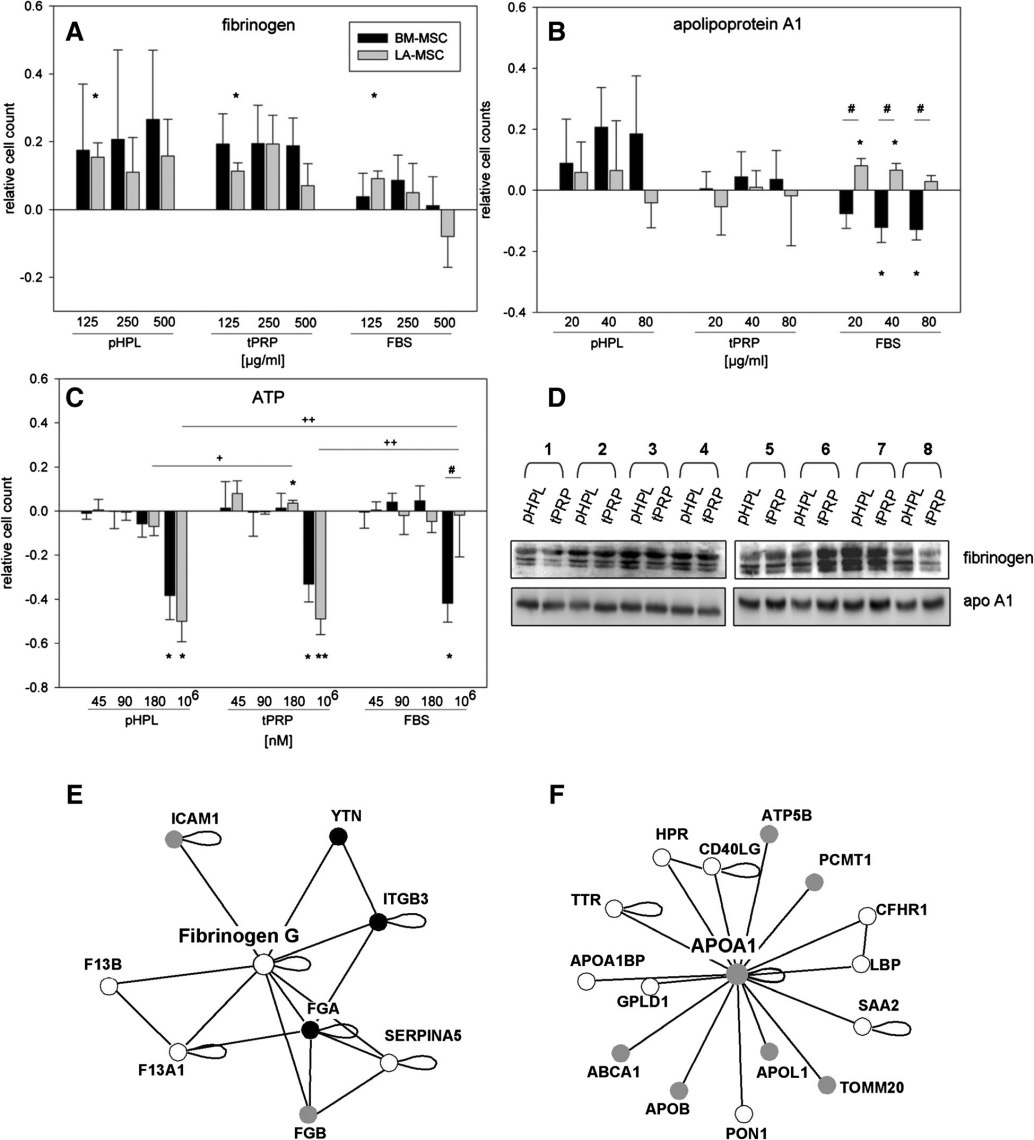
**Influence of identified proteins and ATP on MSC proliferation. (A-C)** MSC were seeded in 5% pHPL, tPRP and FBS, respectively, and stimulated for 3 days with: **(A)** 125, 250 and 500 μg/ml fibrinogen, **(B)** 20, 40 and 80 μg/ml apoA1 and **(C)** 45, 90, 180, and 10^6^ nM ATP. Cell counts were measured using the CellTiter-Glo assay and then normalized to the unstimulated control to calculate relative cell count values. Symbols indicate statistically significant diffences between: * stimulation; + supplements; # MSC sources; (one symbol p < 0.05; two symbols p < 0.01). **(D)** Western blot verification of differentially expressed proteins, fibrinogen and apo-A1 in pHPL and tPRP (2.5 μg protein/lane, eight different batches, respectively). Functional platelet interaction network analysis for fibinogen gamma **(E)** and apolipoprotein A1 **(F)** was performed using PlateletWeb and subnet extraction according to Boyanova et al. [20] (white circles denote platelet proteins with no detected phosphorylation sites; black circles denote platelet proteins with phosphorylation sites detected in platelets; grey circles denote platelet proteins with phosphorylation sites detected in human cells).

First, the 2D-DIGE results were verified by SDS-PAGE and Western blot analysis in ∅ Alb/IgG-samples. Fibrinogen, apoA1, CLIC and also of Annexin A5 were differentially concentrated in ∅ Alb/IgG pHPL and tPRP samples (Figure 2B). Second, all proteins were analyzed in the eight different pHPL and tPRP batches (non-∅ Alb/IgG) used as 10% supplement. CLIC1 was not detectable in the 10% supplemented medium and thus not further analysed. Fibrinogen and apoA1 were non-uniformly concentrated (Figure 3D). To define the concentration of fibrinogen and apoA1, one batch was compared to a dilution series of known concentrations of fibrinogen and apoA1 in SDS-PAGE and Western blotting. By comparing band intensities a concentration of approximately 500 μg/ml fibrinogen in 10% pHPL and approximately 80 μg/ml apoA1 in 10% tPRP-supplemented medium was rated (not shown).

### Influence of selected identified proteins on MSC proliferation

Since none of the identified proteins was yet known as a mitogen for MSC, those described to affect the proliferation of other cell types were further analyzed [26-28]. To assess their bioactivity, BM- and LA-MSC were cultured in 5% pHPL, tPRP and FBS, respectively, plus an increasing concentration of the selected candidate proteins. To reduce variability, pHPL and tPRP batch 7 was chosen for all these experiments. Of note, none of the single candidate proteins was able to affect proliferation in completely supplement-free conditions (not shown).

#### Fibrinogen

125 μg/ml Fibrinogen significantly stimulated LA-MSC proliferation in all three supplements compared to the unstimulated control (Figure 3A). BM-MSC exhibited strong donor variation ranging from stimulation to inhibition especially in pHPL. Fibrinogen can form a matrix to support cell adhesion, migration, but also proliferation [26,29]. It has already been shown to dose-dependently control BM-MSC proliferation, to reduce it at high concentrations depending on the FBS concentration [30,31]. By differential DIGE/MS, we found a differential pattern of fibrinogen species, thus spliced, truncated and posttranslational modified proteins [32,33]. Single batch Western blotting analysis failed to confirm DIGE/MS differential fibrinogen expression. Probably the primary anti-fibrinogen antibody may not be selective for all the individual isoform epitopes identified by DIGE/MS proteomics. The higher concentration of fibrinogen gamma chain species in pHPL well fits to the thrombin-induced conversion of fibrinogen to fibrin, consequently removed as clot in tPRP. By applying platelet-specific systems biology analysis several interaction partners of fibrinogen gamma such as ICAM1 (Intercellular Adhesion Molecule 1) or integrin beta 3 (CD61), known to be involved in rapid platelet aggregation, have been identified (Figure 3E).

#### Apolipoprotein A1

Only in FBS MSC proliferation became significantly affected by ApoA1, which selectively promoted LA-MSC but inhibited BM-MSC proliferation (Figure 3B). These observations illustrate the differences in MSC tissue source and supplements, respectively. ApoA1, a known major component of high density lipoprotein (HDL), has been reported to promote proliferation and to inhibit apoptosis of endothelial and vascular smooth muscle cells and to induce cardiac differentiation of embryonic stem cells [27]. Several functional interaction partners like Apo A1 binding protein and lipopolysaccharide binding protein are depicted in Figure 3F after performing platelet-specific systems biology analysis [20].

#### ATP

Apart from the proteins identified by the proteomic approach, the ATP-based CellTiter-Glo assay indicated higher ATP concentrations in tPRP supplement (6/8 batches; 176.5 ± 110.7 nM in tPRP and 52.3 ± 34.9 nM in pHPL). Extracellular ATP (eATP) has been described to affect various cellular features such as proliferation, apoptosis and arrest of growth, especially in neural cells [34]. In MSC, spontaneously released ATP has been associated with decreased proliferation, reversible by P2 and P2Y1 antagonists [35]. Whereas this inhibitory effect of high ATP concentrations of 10^6^ nM was reproducible in all three supplements for BM-MSC [35,36], LA-MSC in FBS appeared refractory to growth inhibition (Figure 3C). The distinct effects of eATP on different cell types have been attributed to engagement of different purinergic receptors [37,38].

Besides the current experimental limitations (depletion of abundant proteins, no membrane proteins expected, whole protein-based proteomics, selected pH range 4–7 of IEF), we are confident that the combination of differential proteomics, database comparison and functional testing proves to be a suitable method to identify relevant factors affecting MSC proliferation. Despite the few factors tested, we were able to identify some with differential effects on MSC proliferation depending on the supplement and MSC tissue source. Thus, the major hurdle in developing a chemically-defined medium is the combination and interaction of different factors which can best be modeled by multifactorial design experiments [39].

### Influence of selected reference cytokines on MSC proliferation

**Figure 4:**
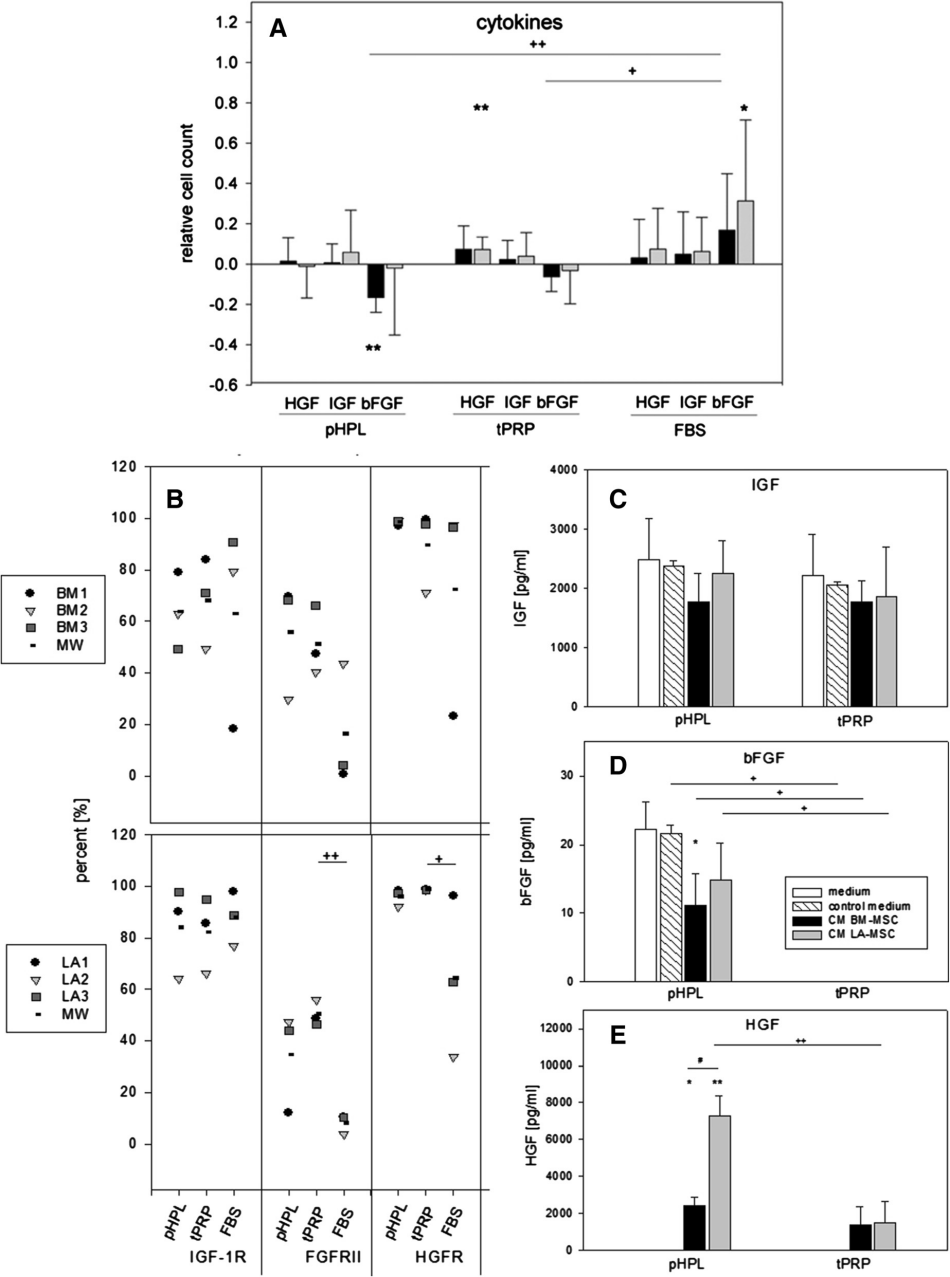
**Influence of selected cytokines on MSC proliferation; receptor expression profiles and concentration. (A)** MSC were seeded in 5% pHPL, tPRP and FBS, respectively, and stimulated with for 3 days with 50 ng/ml HGF, 10 ng/ml IGF-1 or 25 ng/ml bFGF. Cell counts were acquired with the CellTiter-Glo assay and then normalized to the unstimulated control to derive relative cell count values. **(B)**% positivity of IGF, FGF and HGF receptor expression of BM- and LA-MSC (donors 1–3, respectively) in pHPL, tPRP and FBS assessed by flow cytometry. **(C – E)**: IGF, FGF and HGF concentrations were determined by ELISA in pHPL and tPRP supplemented medium (medium, 6 different batches); medium stored for 24 h (control medium) and conditioned by MSC (CM) (each n = 3). Symbols indicate statistically significant diffences between: * stimulation; + supplements; # MSC sources; (one symbol p < 0.05; two symbols p < 0.01).

Cytokines were not identified within the current 2D DIGE approach probably due to their often low concentration in plasma. Therefore, within a second set of experiments selected cytokines, known to foster MSC proliferation in FBS [40-42], were added to the different supplements and MSC proliferation was measured. Here we a) tested the effect of adding different concentrations of cytokines, b) evaluated growth factor receptor expression and c) the concentration of cytokines in the culture medium/conditionened medium. We decided to use 50 ng/ml HGF, 10 ng/ml IGF-1 and 25 ng/ml bFGF [35,40,41]. Cytokines failed to induce a proliferative response in MSC cultured with 5% platelet derivatives (Figure 4A), whereas control cells in 5% FBS responded adequately. bFGF inhibited BM-MSC proliferation in the human supplements. Comparable findings have been observed by Cheon et al. testing the effects of EGF, FGF and ITS (insulin, transferring, selenium) on AT-MSC proliferation in FBS or human serum. In both settings, the combination of ITS and growth factors induced proliferation, whereas FGF on its own was highly effective in FBS only [43]. Addition of EGF, bFGF and PDGFbb to 3% platelet poor plasma, however, reached a proliferation rate comparable to FBS [44], advising a fine tuning in multifactorial design studies.

Since cytokine effects depend on their concentration as well as the receptor expression, flow cytometry analysis was performed to assess expression of growth factor receptors. FGFRII and HGFR were expressed at a significantly lower number of LA-MSC in FBS (similar, but not significant in BM-MSC) (Figure 4B). Interestingly, bFGF only induced proliferation in FBS, where both MSC types had the lowest proportion of cells expressing FGFRII (approx. 10%). The higher proportion of FGFRII expression in the human supplements possibly rendered MSC unresponsive to additional bFGF. PDGFR-α expression appeared to be slightly higher in FBS, whereas PDGFR-β and TGF-βRII were expressed similar to FGFRII and HGFR (Additional file 3: Figure S3). With the exception of PDGFR-β in FBS, no differences between BM- and LA-MSC were observed.

In previous studies, we addressed the cytokine content in pHPL, tPRP and conditioned media of MSC but failed to explain differential effects of pHPL and tPRP on MSC proliferation [13]. We now further analyzed the levels of selected candidates, including HGF, IGF-1, bFGF, PDGF-AA, AB and BB and TGF-βII in fresh medium with 10% supplement (medium), in medium incubated for 24 h without cells (control medium) or in medium conditioned by MSC (conditioned medium, CM). TGF-βII was not detectable in any of the samples. IGF-1 concentrations remained largely unaffected in all conditions (Figure 4C). bFGF was detectable only in the pHPL setting, significantly diminished by BM-MSC (Figure 4D). HGF was only detectable in CM with apparent differences between BM- and LA-MSC in pHPL, but not in tPRP (Figure 4E). PDGF-AA levels were comparable in pHPL at all four conditions, but in tPRP values dropped in control medium to yield significant differences compared to pHPL. PDGF-AB levels, although initially comparable between pHPL and tPRP, became strongly reduced in tPRP control medium. Similar data were obtained for PDGF-BB, with differences in pHPL CM between BM- and LA-MSC (Additional file 3: Figure S3).

Individual experimental conditions such as donor variability, but obviously also the MSC tissue origin, can largely affect the outcome as indicated in this study. Reported concentrations of cytokines in the supplements vary enormously and so do the concentrations used to stimulate MSC [45]. Numerous data indicate a strong positive effect of bFGF on MSC proliferation and osteogenic differentiation, either alone or in combination with TGF-β1 [40,46]. Platelet lysate-induced BM-MSC proliferation can be inhibited by 75% by combining neutralizing antibodies against PDGF-AB/BB, TGF-β1 and bFGF. Stimulation with these three factors alone, however, was insufficient to promote proliferation [45]. This is in line with the data presented herein.

## Conclusion

Human supplements, such as platelet derivatives, are emerging as alternatives to FBS in clinical-scale MSC manufacturing. Observed differences in the proliferative response of BM- and LA-MSC towards platelet lysate versus platelet releasate prompted us to initiate a proteomic screen of differentially expressed proteins. Although the identified differential proteins have not been previously shown to affect MSC expansion, bioactivity testing confirmed differential effects of identified proteins and selected cytokines on BM- and LA-MSC in the context of supplement (FBS vs pHPL vs tPRP).

We are confident that combining already existing data on MSC mitogens and platelet and plasma proteome databases with the current data on identified proteins and their differential bioactivity will provide a platform to identify novel factors to optimize and standardize BM- and LA-MSC culture conditions for safer clinical application.

## Methods

The work involving human material has been carried out in accordance with *The Code of Ethics of the World Medical Association (Declaration of Helsinki) for experiments involving humans*. Use of human material has been approved by the Medical Ethical Committee II in Mannheim. Informed consent has been obtained prior to cells/tissue collection.

### Preparation of platelet derivates: pHPL and tPRP

Buffy coat-derived pooled platelet concentrates (pools of four healthy blood donors, all fulfilling the criteria for blood donor eligibility), were prepared in AB-plasma from one donor [13]. To further minimize donor variability, each batch was prepared by pooling two platelet concentrates. To assess variability between batches, eight different pooled batches have been analyzed.

Each batch was then split into two parts. To yield the whole platelet lysate pHPL, one part was processed by a freezing and thawing step at −30°C and 37°C. The other part was activated with 100U/ml thrombin from human plasma (Merck) for 60 min under gentle agitation to derive the releasate tPRP, containing only the released platelet proteins in plasma. Here, in contrast to the pHPL, a fibrin clot was formed and depleted by centrifugation affecting the relative composition of platelet and plasma factors compared to pHPL. Further processing was standardized for both derivatives: samples were centrifuged at 2000 g, 20 min at RT, supernatant was aliquoted and cryopreserved at −30°C. Prior to use, derivatives were gently thawed and centrifuged at 2000 g for 10 min. To prevent clotting, DMEM medium (Lonza, supplemented with 100 U/ml penicillin, 0.1 mg/ml streptomycin and 4 mM L-glutamine (all from PAA) was added with 5 IU/ml heparin (Ratiopharm). The respective supplement was added at the indicated concentration and then the medium filtered through a 0.45 μm filter. pHPL- and tPRP was always prepared fresh and used within 24 hours.

### Isolation and expansion of MSC

Adipose tissue was collected from three female donors undergoing elective tumescence liposuction of the abdomen and hip/thigh region. BM aspirates were obtained from three healthy donors in the department of Internal Medicine V at the University Hospital of Heidelberg by puncturing the iliac crest. LA-MSC and BM-MSC were isolated by collagenase digestion or density gradient centrifugation (Ficoll-Paque Plus =1.078 g/ml, GE Healthcare) respectively as described previously [10,11,13,47]. Primary cells were seeded in 10% pHPL, tPRP and FBS (PromoCell), respectively. At a confluence of 70-80%, cells were passaged using trypsin-EDTA (PAA) and either replated at a density of 200 cells per cm^2^ for all passages or used for experiments. MSC were characterized regarding the proliferation capacity, immune phenotype, adipo- and osteogenic differentiation potential (Additional file 1: Figure S1), according to previously described methods [11,13]. Chondrogenic differentiation was not performed.

### Differential proteomics

#### Sample preparation

To deplete abundant proteins, pHPL and tPRP, each pooled from two batches (a total of 16 donors and plasma from 4 donors), were pretreated with Albumin/IgG Depletion Kit (GE Healthcare; ∅ Alb/IgG) according to the manufacturer’s instruction (Figure 1A). Samples were mixed with prefrozen acetone/methanol (v/v 8:1) for 3 h at −30°C and subsequently centrifuged at 10000 g for 1 h at 4°C. After washing with 500 μl ice-cold acetone and centrifugation (10000 g, 10 min, 4°C), the pellet was air dried and resuspended in rehydration buffer (6 M urea, 2 M thiourea, 2% CHAPS (all Roth), 0.002% bromophenol blue (Sigma-Aldrich).

### Two-dimensional difference gel electrophoresis

To separate, quantify and identify differential protein signatures and protein species in ∅ Alb/IgG pHPL and in ∅ Alb/IgG tPRP, multiparametric 2D-DIGE (two-dimensional difference gel electrophoresis) was performed [32,33,48,49]. Thus, 50 μg protein of each sample was labelled with CyDye DIGE Fluors (Cy3, Cy5, respectively, GE Healthcare) according to the Minimal labeling protocol (GE Healthcare). For an internal standard, equal amounts of pHPL and tPRP were mixed and 50 μg from this mix labelled with Cy2. Labelled platelet derivate samples and internal standard were pooled equally to a final protein quantity of 150 μg. Rehydration buffer with 32 mM DTT (Roth) and 0.5% IPG buffer (pH 4–7, BioRad) was added to a final volume of 450 μl. After 24 h rehydration, the 24 cm IPG-strips (pH 4–7, BioRad) were subjected to isoelectric focussing on an IPGphor (GE Healtcare) for a total of 58000 V/h. This specific range of pH (pH 4–7) was chosen, since most of the proteins seem to occur in this isoelectric window, after testing IPG-strips with a range from pH 3–10 (data not shown). Then each strip was equilibrated in 15 ml equilibration buffer (6 M urea, 30% glycerol, 2% SDS, 50 mM Tris (pH 8.8), 0.002% bromophenol blue, all Serva), first with 32 mM DTT for 20 min, second with 135 mM iodoacetamide (Roth). Equilibrated strips were transferred onto a homogenous precast 12% bis-tris polyacrylamide gel (1 mm, Serva) and sealed with agarose (1% agarose, 0.002% bromophenol blue, Roth). SDS-PAGE was performed with constant 17 W per gel and a running time until the blue front ran out of the gels (Ettan DALT II system; GE Healthcare). Three replicates were run.

#### Advanced image warping and quantitative protein analysis

Visualization of labelled protein spots was performed with a fluorescence scanner (FujiFilm FLA-5100). Therefore, gels were scanned with three different fluorescence filters (Cy2: Ex. 488 nm, Em. 520 nm, Filter Y520/LBB; Cy3: Ex. 532 nm, Em. 580 nm, Filter O580/DGR1; Cy5: Ex. 633 nm, Em. 670 nm, Filter R665/LPR). Sensitivity was set on 400 V with 50 μm pixel/resolution. Spot detection, warping, and statistical analysis of stained or labeled samples were performed using Delta2D 3.4 software (Decodon) as described previously [50]. For significant differential protein spot detection, sensitivity, background settings, and spot size were chosen according to the manufacturer’s instruction. Images were matched using stringent warping strategy. Regulation factor was set to 1 +/− 0.5 (red spots >1.5; green spots <0.8; and yellow spots <1.5, >0.8).

#### Mass spectrometry and protein identification

Preparative gels were run (250 μg protein/gel), fixed with methanol/acetic acid/H_2_O (40/10/50%), stained with Sybro Ruby (GE Healthcare) and scanned at 618 nm. In addition, a sensitive coomassie G250 blue staining was performed. Matched protein spots of interest were picked manually and subjected to an in-gel tryptic digestion as described previously [51]. Briefly, after washing (100 μl H_2_O, 8 min, 37°C, 600 rpm), picked spots were incubated in 200 μl 40 mM NH_4_HCO_3_/CAN (acetonitrile) (v/v 1:1) for 15 min (25°C, 600 rpm). After incubation with ACN (1 min, 600 rpm, RT), protein spots were air dried for 5 min. For peptide mass fingerprint mapping 10 μl trypsin (3.5 μg/μl, Promega) was added for 30 min at 4°C. Excessive trypsin was removed and 10 μl 40 mM NH_4_HCO_3_ added and incubated over night at 37°C. Samples were stored at −30°C.

Before MALDI analyses of digested human platelet proteins, peptides were concentrated and manually desalted using ZipTip® pipette C18 columns (Millipore). Peptides were eluted with 1 mL α-cyano-4-hydroxycinnamic acid diluted 1:7 in 1:1 ACN/0.1% trifluoracetic acid solution and spotted on a MALDI 800–384 anchor chip target plate and analyzed automatically on a Bruker Ultraflex II using flex control, flex analysis, and Bruker BioTools software (batchmode). For external calibration, peptide calibration standard II was used (Sigma; m/z ratio from 700 to 3500) [50]. Database searches were performed using MS ion search against all entries for Homo sapiens in the Swiss-Prot database, applying the following: tryptic digest, up to one missed cleavage; fixed modifications, carbamidomethyl (C); variable modification, and oxidation at methionine (M). Mass tolerance was set to 7100 ppm. Identification was taken as unambiguous if the Mowse score was at least 56 (with p-value < 0.05). In addition, PMF identification was verified comparing molecular weight and pI of the identified protein with gel position of the according protein spot.

#### Data verification by Western Blot analysis

Proteins were lysed in RIPA buffer (Santa Cruz Biotech). Protein concentration was measured by Bradford protein assay (Bio-Rad) and adjusted to equal concentrations. Protein lysates were mixed with loading buffer (Fermentas), separated on 12% Bis-Tris gels and then transferred onto a PVDF membrane (GE Healthcare). Primary antibodies used were: α-Fibrinogen-Biotin pAB (IMS06-038-312, Agrisera), α-Apolipoprotein A1 pAB (178422, Calbiochem), α-CLIC-1 (chloride intracellular channel 1; 356.1, Santa Cruz Biotech). HRP-conjugated secondary reagents used were α-Mouse IgG, α-Rabbit IgG (both Dako) and Streptavidin (Calbiochem). All antibodies were dissolved in TBS, 5% skimmed milk powder, 0.05% Tween-20 (all Roth). Band detection was performed using enhanced chemiluminescence reagent (Amersham™ ECL™ Select Western Blotting Detection Reagent, GE Healthcare). Loading concentrations were assessed by Ponceau S staining and reference controls (Serva dual color protein standard III, Serva).

### Proliferation and stimulation assay

To determine the proliferation rate of MSC, CellTiter-Glo™ assay (Promega) quantifying ATP was performed. MSC in passage 2 with 70% confluence were trypsinized and seeded at a density of 3000 cells for 3 days into a black 96 well plate (Iso plate, PerkinElmer). Culture medium was either supplemented with each 10%, 7.5%, 5%, 2.5% pHPL, tPRP or FBS, respectively. Stimulation experiments utilized 5% of the supplements and the following substances at the indicated concentrations: fibrinogen, apolipoprotein A1 (apoA1), adenosine triphosphate (ATP) (Roche) and in a second set of experiments hepatocyte growth factor (HGF), insulin-like growth factor 1 (IGF-1) (all Calbiochem), and basic fibroblast growth factor (bFGF) (Miltenyi Biotec).

### Enzyme-linked immunosorbent assay (ELISA)

In order to detect cytokines, described to induce MSC proliferation, platelet derivates (pHPL, tPRP n = 6; FBS n = 2) as well as medium of MSC (BM-, LA-MSC in FBS, pHPL, tPRP, each n = 3), conditioned for 24 h, were tested for PDGF-AA, AB, BB; IGF-1; HGF, bFGF (all Quantikine ELISA kit, R&D Systems) by ELISA according to the manufacturer’s instructions.

### Flow cytometry

To assess the expression of cytokine receptors, flow cytometric analyses were performed. MSC were double-stained in 100 μl cell wash (BD) with the following phycoerythrin- (PE) and allophycocyanin- (APC) labelled antibodies: PDGFR-α-PE (16A1), PDGFR-β-APC (18A2, both biolegend), TGF-βRII-PE (FAB2411P; R&D systems), IGFR-1-APC (1H7; eBioscience), HGFR-APC (FAB35821), FGFR-PE (FAB684P; both R&D systems). 7-Aminoactinomycin D was used for dead cell exclusion (Beckman Coulter). Stained cells were analyzed with the FACS Canto II from BD.

### Statistical analysis

Statistical tests were performed using SigmaPlot 11.0 (Systat Software Inc). Data was tested for normality and equal variance before analysis. Statistical differences between three samples were calculated using analysis of variance (ANOVA, Holm-Sidak method; or ANOVA on ranks if equal variance testing failed, Tukey method). Comparing two samples paired or unpaired t-tests, respectively, were used. The symbols indicate: (*) significant difference before and after stimulation; (+) significant difference between supplements; (#) significant difference between MSC sources.

## Abbreviations

ACN: Acetonitrile; APC: Allophycocyanin; apoA1: Apolipoprotein A1; ATP: Adenosine triphosphate, bFGF, Basic fibroblast growth factor; BM-MSC: Bone marrow-derived mesenchymal stromal cells; CLIC-1: Chloride intracellular channel 1; CM: Conditioned medium; Cy: CyDye; FGFRII: Fibroblast growth factor receptor II; GMP: Good manufacturing practice; HGF: Hepatocyte growth factor; HGFR: Hepatocyte growth factor receptor, c-met; IGF-1: Insulin-like growth factor 1; IGFR-1: IGF-1 receptor 1; LA-MSC: Lipoaspirate-derived MSC, adipose tissue-derived MSC; MSC: Mesenchymal stromal cells; PDGF-AA, AB, BB: Platelet-derived growth factor; PDGFR-αβ: PDGF receptor; PE: Phycoerythrin; pHPL: Pooled human platelet lysate; TGF-βΙΙ: Transforming growth factor beta II; TGF-βRII: TGF-β receptor II; tPRP: Thrombin-activated platelet releasate in plasma; ØAlb/IgG: Albumin and IgG depleted.

## Competing interests

The authors declare that they have no competing interests.

## Authors’ contributions

SK carried out the whole experiments, including the proteomic analysis, immunoassays and functional testing, performed statistical analysis and drafted the manuscript. LD participated in the design of the study, carried out the evaluation and statistical analysis of proteomic data sets. HK participated in the design of the study and interpretation of results. HJT participated in the conception and coordination of the study, was involved in the statistical analysis and interpretation of data and drafted the manuscript. KB conceived of the study, participated in the design and analysis of experiments and coordinated the study. She drafted the manuscript. All authors proof read the manuscript and approved the final version.

## Authors’ information

SK: was a PhD student and well experienced in protein analysis, including proteomics and immunoblotting.

LD: was a PostDoc in HJTs laboratory and well experienced in proteomic analysis including mass spectometry.

HK: is head of the Institute of Transfusion Medicine and Immunology and experienced with all aspects of blood banking and transfusion services.

HJT: was head of the Laboratory of Immunology & Proteomics, Department of Dermatology and University Medical Center Mannheim, Heidelberg University, Germany; has moved to the Unit Experimental Research –Immunology & Proteomics– at the German Federal Institute for Risk Assessment.

KB: is head of the stem cell research department within the institute and well experienced in the field of MSC and defining human supplements to replace fetal bovine serum for GMP-compliant manufacturing processes.

## Supplementary Material

Additional file 1: Figure S1 Phenotype, differentiation potential and immunosuppressive activity LA- and BM-MSC were cultured in 10% FBS, pHPL and tPRP, respectively. (A) Photos were taken at identical time points post-seeding to observe cell morphology and confluence. Representative pictures of one LA-MSC and one BM-MSC batch are depicted (all 100× magnification). (B) Adipogenic and osteogenic differentiation. Cells were induced with differentiation media (adipogenic induction and maintance medium for 3 and 4 days, respectively and osteogenic induction medium, all Lonza) for 3 weeks and then stained with oil red o and van Kossa as descried previously [11,13]. Representative results of one LA-MSC batch are depicted (100× magnification). (C) Immunosuppressive activity was assessed by coculturing LA-MSC (ratio 1:10) with allogeneic peripheral blood leukocytes labeled with carboxyfluorescein diacetate succinimidyl ester (5 μM, Vybrant CFDA-SE cell tracker kit, Invitrogen). Leukocyte proliferation was stimulated with phytohemagglutinin (2.5 μg/ml PHA-L, Roche Applied Science) and assessed by progressive halving of CFDA-SE fluorescence (red line – control with PHA; green – control without PHA). One representative experiment is depicted revealing similar dye retention and thus immunosuppressive activity of MSC in FBS, pHPL and tPRP (grey line – coculture MSC with PHA). Similar data were obtained applying BM-MSC. Click here for file

Additional file 2: Figure S2 Representative 2D-gel of pHPL and PRP samples. Spots marked in green occurred at higher concentrations in tPRP as compared to pHPL, whereas spots marked in red were more abundant in pHPL (pH 4–7; 24 cm strip; Sybro Ruby staining). Click here for file

Additional file 3: Figure S3 Cytokines and receptor expression. (A) Flow cytometry to determine PDGFs and TGF-βII receptor expression (% positivity) of BM- and LA-MSC in pHPL, tPRP and FBS. (B – D) Determination of PDGF-AA, AB, BB concentrations in six different pHPL and tPRP batches (10% supplemented medium); medium stored for 24 h (control medium) and conditioned medium by MSC (CM) via ELISA. Symbols indicate statistically significant diffences between: * stimulation; + supplements; # MSC sources; (one symbol p < 0.05; two symbols p < 0.01; n = 3 for each condition). Click here for file
