# Supplementary figures and images for: Functional and differential proteomic analyses to identify platelet derived factors affecting ex vivo expansion of mesenchymal stromal cells
Source: BMC Cell Biol. 2013 Oct 30;14:48. doi: 10.1186/1471-2121-14-48 (PMC4231358; doi:10.1186/1471-2121-14-48)

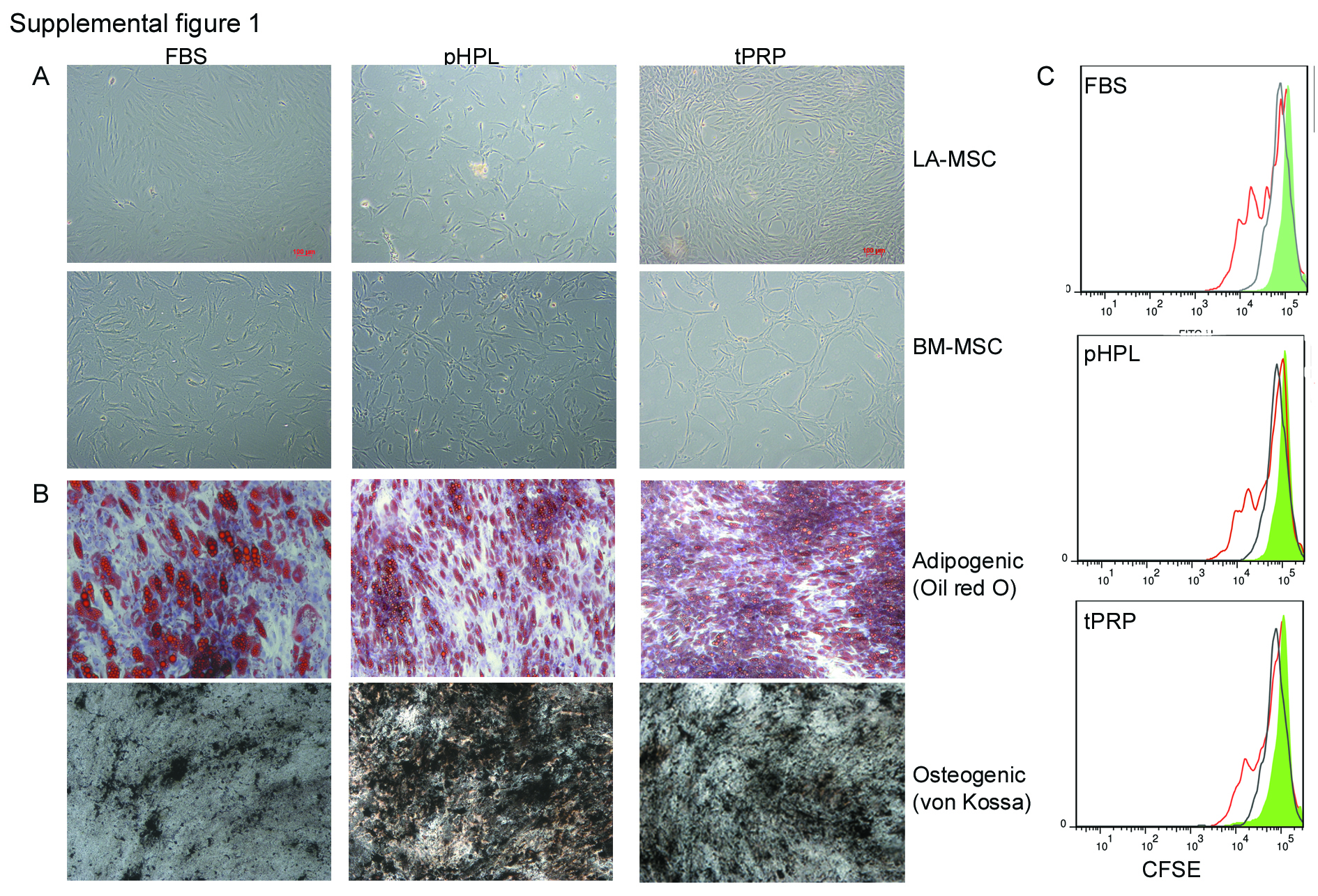

Supplement: Additional file 1: Figure S1 — Phenotype, differentiation potential and immunosuppressive activity LA- and BM-MSC were cultured in 10% FBS, pHPL and tPRP, respectively. (A) Photos were taken at identical time points post-seeding to observe cell morphology and confluence. Representative pictures of one LA-MSC and one BM-MSC batch are depicted (all 100× magnification). (B) Adipogenic and osteogenic differentiation. Cells were induced with differentiation media (adipogenic induction and maintance medium for 3 and 4 days, respectively and osteogenic induction medium, all Lonza) for 3 weeks and then stained with oil red o and van Kossa as descried previously [11,13]. Representative results of one LA-MSC batch are depicted (100× magnification). (C) Immunosuppressive activity was assessed by coculturing LA-MSC (ratio 1:10) with allogeneic peripheral blood leukocytes labeled with carboxyfluorescein diacetate succinimidyl ester (5 μM, Vybrant CFDA-SE cell tracker kit, Invitrogen). Leukocyte proliferation was stimulated with phytohemagglutinin (2.5 μg/ml PHA-L, Roche Applied Science) and assessed by progressive halving of CFDA-SE fluorescence (red line – control with PHA; green – control without PHA). One representative experiment is depicted revealing similar dye retention and thus immunosuppressive activity of MSC in FBS, pHPL and tPRP (grey line – coculture MSC with PHA). Similar data were obtained applying BM-MSC. [file 1471-2121-14-48-S1.jpeg]

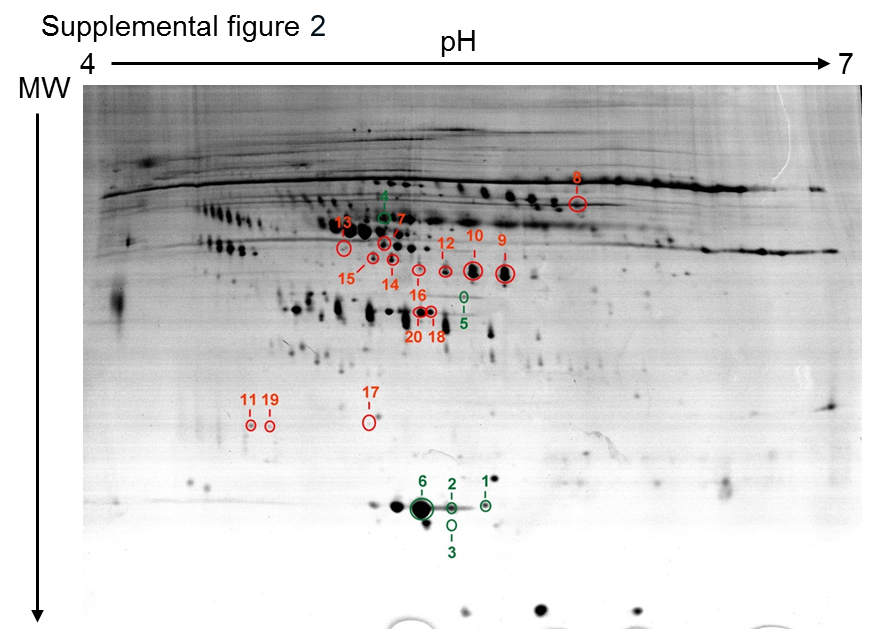

Supplement: Additional file 2: Figure S2 — Representative 2D-gel of pHPL and PRP samples. Spots marked in green occurred at higher concentrations in tPRP as compared to pHPL, whereas spots marked in red were more abundant in pHPL (pH 4–7; 24 cm strip; Sybro Ruby staining). [file 1471-2121-14-48-S2.jpeg]

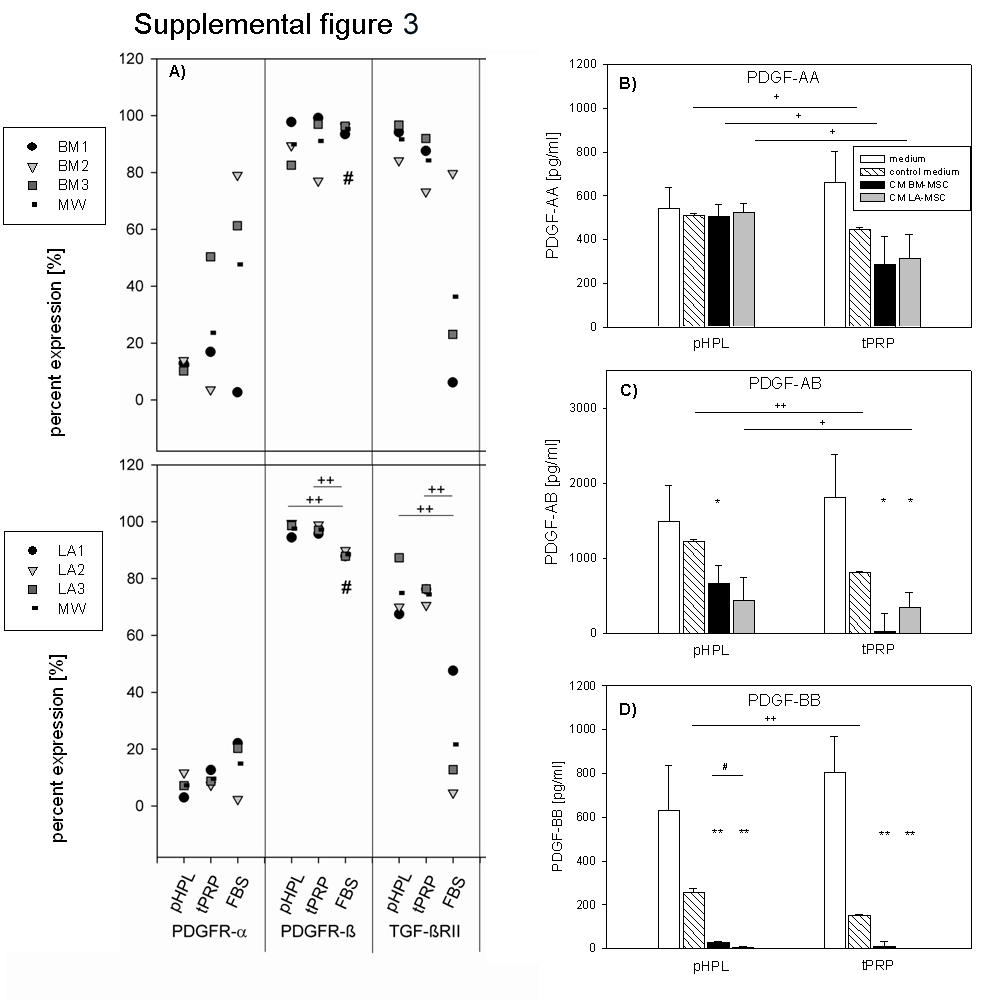

Supplement: Additional file 3: Figure S3 — Cytokines and receptor expression. (A) Flow cytometry to determine PDGFs and TGF-βII receptor expression (% positivity) of BM- and LA-MSC in pHPL, tPRP and FBS. (B – D) Determination of PDGF-AA, AB, BB concentrations in six different pHPL and tPRP batches (10% supplemented medium); medium stored for 24 h (control medium) and conditioned medium by MSC (CM) via ELISA. Symbols indicate statistically significant diffences between: * stimulation; + supplements; # MSC sources; (one symbol p < 0.05; two symbols p < 0.01; n = 3 for each condition). [file 1471-2121-14-48-S3.jpeg]
